# Andrographolide attenuates LPS-stimulated up-regulation of C-C and C-X-C motif chemokines in rodent cortex and primary astrocytes

**DOI:** 10.1186/s12974-016-0498-6

**Published:** 2016-02-09

**Authors:** Siew Ying Wong, Michelle G.K. Tan, William A. Banks, W.S. Fred Wong, Peter T.-H. Wong, Mitchell K.P. Lai

**Affiliations:** Department of Pharmacology, Yong Loo Lin School of Medicine, National University of Singapore, 16 Medical Drive, Kent Ridge, 117600 Singapore; Department of Clinical Research, Singapore General Hospital, Outram, Singapore; Geriatric Research Education and Clinical Center, Veterans Affairs Puget Sound Health Care System, Seattle, WA USA; Department of Medicine, Division of Gerontology and Geriatric Medicine, University of Washington School of Medicine, Seattle, WA USA; Immunology Program, Life Science Institute, National University of Singapore, Kent Ridge, Singapore

**Keywords:** Andrographolide, Cytokines, Chemokines, Astrocyte, Neuroinflammation

## Abstract

**Background:**

Andrographolide is the major bioactive compound isolated from *Andrographis paniculata*, a native South Asian herb used medicinally for its anti-inflammatory properties. In this study, we aimed to assess andrographolide’s potential utility as an anti-neuroinflammatory therapeutic.

**Methods:**

The effects of andrographolide on lipopolysaccharide (LPS)-induced chemokine up-regulation both in mouse cortex and in cultured primary astrocytes were measured, including cytokine profiling, gene expression, and, in cultured astrocytes, activation of putative signaling regulators.

**Results:**

Orally administered andrographolide significantly attenuated mouse cortical chemokine levels from the C-C and C-X-C subfamilies. Similarly, andrographolide abrogated a range of LPS-induced chemokines as well as tumor necrosis factor (TNF)-α in astrocytes. In astrocytes, the inhibitory actions of andrographolide on chemokine and TNF-α up-regulation appeared to be mediated by nuclear factor-κB (NF-κB) or c-Jun N-terminal kinase (JNK) activation.

**Conclusions:**

These results suggest that andrographolide may be useful as a therapeutic for neuroinflammatory diseases, especially those characterized by chemokine dysregulation.

**Electronic supplementary material:**

The online version of this article (doi:10.1186/s12974-016-0498-6) contains supplementary material, which is available to authorized users.

## Background

Chemokines are small (8–12 kDa), chemotactic proteins with multiple functions. In the central nervous system (CNS), chemokines mediate innate and adaptive immune responses, signaling for leukocyte recruitment as well as glial cell activation during neuroinflammation [1]. Furthermore, they are known to play important roles in interneuronal communication, neuronal progenitor cell migration, and oligodendrocyte maturation [2–4]. Chemokines are divided into four subfamilies, namely C-C, C-X-C, C-X3-C, and C based on the relative positions of conserved cysteine residues in the N terminus [5]. Among the subfamilies, the C-C and C-X-C chemokines comprise the two largest groups [5]. In response to interleukin (IL)-1β or tumor necrosis factor (TNF)-α released during the acute phases of pathogen invasion or tissue injury, multiple cytokines/chemokines are up-regulated, then secreted by cells (primarily microglia and astrocytes) of the brain parenchyma [6, 7]. While cytokines/chemokines help clear harmful stimuli by initiating inflammatory responses and recruiting peripheral immune cells, dysregulation of chemokines has been implicated in various neurological diseases characterized by neuroinflammation, including neurodegenerative dementias such as Alzheimer’s disease, multiple sclerosis, traumatic brain injury, and specific types of meningitis [8–11]. Therefore, there is an unmet need for safe and potent anti-neuroinflammatory therapeutics.

Andrographolide is a bioactive labdane diterpenoid derived from the herbaceous *Andrographis paniculata*, which has been traditionally used in Asia to treat a variety of ailments, including fever, cough, tuberculosis, snake bites, and respiratory or urinary tract infections [12, 13]. Andrographolide has been found to have anti-cancer, anti-bacterial, anti-inflammatory, and anti-oxidative effects in target organs such as liver, lung, and bladder [14–18], where the molecules are purported to permeate cells via passive diffusion [19], as a andrographolide-specific receptor has not been described as yet. The extracts of *A. paniculata*, whose pharmacological efficacy is attributed mainly to andrographolide [13], have been used in clinical trials for various inflammatory conditions [20, 21]. In contrast, there are few studies which assessed the anti-neuroinflammatory effects of andrographolide. We and others have previously reported anti-inflammatory and neuroprotective effects of andrographolide which seem to be mediated via regulation of microglia as well as astrocytes [22, 23]. We have also shown that andrographolide attenuated IL-1β-stimulated up-regulation of the C-C motif ligand 5 (CCL5) chemokine via nuclear factor-κB (NF-κB) inhibition in vitro [24]. However, it is unclear whether this effect applies to other chemokines, whether it is effective in animals using more disease-relevant models such as bacterial lipopolysaccharide (LPS) endotoxin injection or whether it may involve other signaling molecules besides NF-κB. For example, c-Jun N-terminal kinase (JNK)-mediated signaling is known to be involved in neuroinflammation, and andrographolide has been reported to activate JNK in various cell types [25–28], but it is not clear whether JNK regulation mediates the effects of andrographolide on neuroinflammation. Furthermore, there is increasing recognition that astrocytes play an integral role in regulating neuroinflammatory responses but are less well studied compared to microglia [29–31]. In this study, we first examined the effects of andrographolide treatment on the cortical chemokine levels of mice subjected to LPS injections. Next, we studied changes in expression of a range of chemokines and activation of signaling molecules in LPS/andrographolide-treated rat primary astrocytes.

## Methods

### Reagents, rodent treatment, and brain tissue processing

**Fig. 1:**
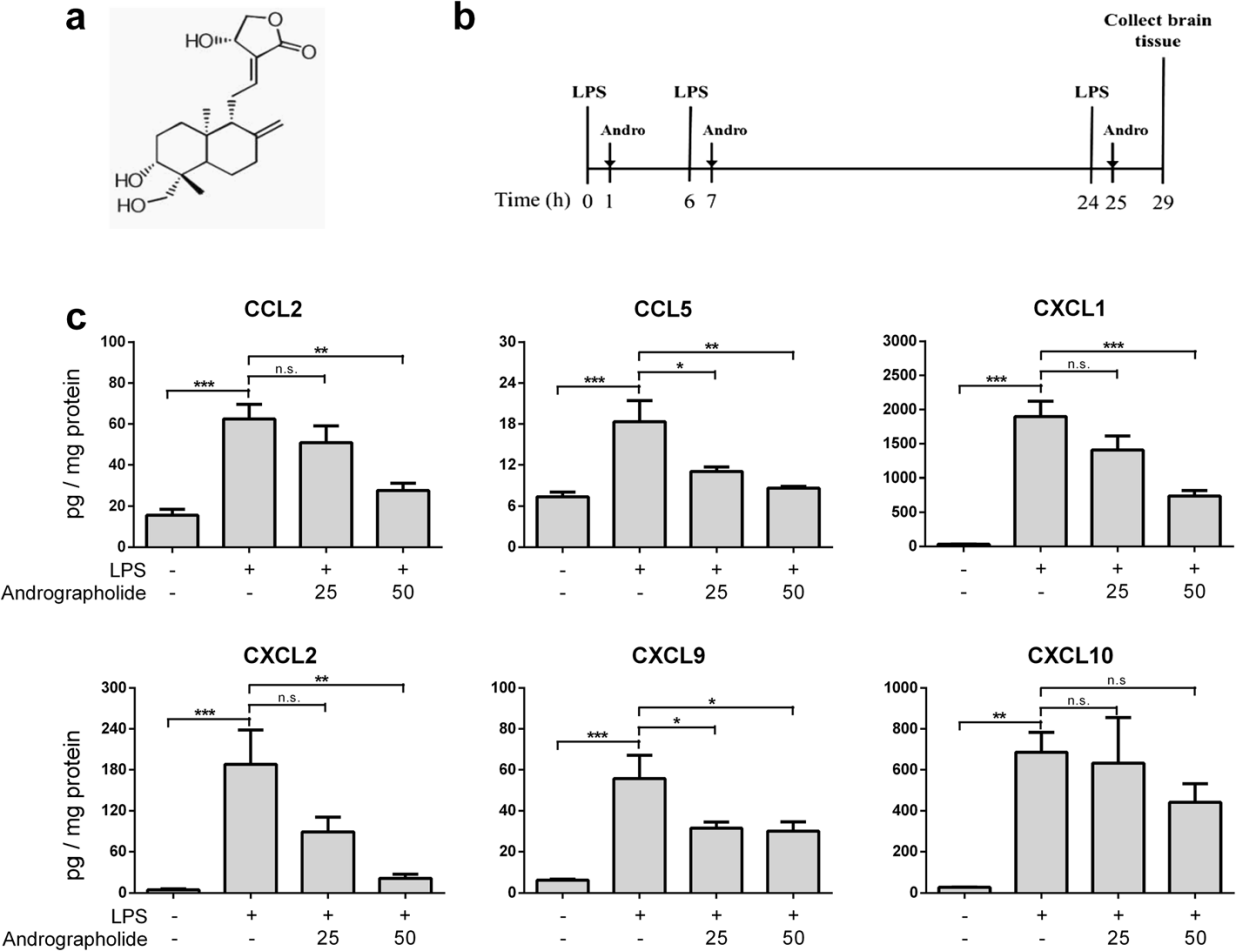
Oral andrographolide inhibited LPS-mediated C-C and C-X-C chemokines in mouse cortex. **a** Structure of andrographolide: 3-[2-[decahydro-6-hydroxy-5-(hydroxymethyl)-5,8a-dimethyl-2-methylene-1-napthalenyl]ethylidene]dihydro-4-hydroxy-2(3H)-furanone (CAS no. 5508-58-7). **b** Mouse LPS treatment regimen with three intraperitoneal LPS injections (3 mg/kg per injection) over 24 h, with andrographolide (25 or 50 mg/kg) administered by oral gavage 1 h after each LPS injection, and animals sacrificed 5 h after the last injection for collection of brain. **c** *Bar graphs* showing mean ± SEM concentrations of various chemokines (in pg/mg total brain protein) in the cortex of control mice (injections with PBS and gavage with PEG vehicle); LPS only (LPS injections and gavage with vehicle); LPS + 25 mg/kg andrographolide; and LPS + 50 mg/kg andrographolide (*N* = 7–9 animals per group). *p < 0.05; **p < 0.01; ***p < 0.001. *n.s.* not significant (*p* > 0.05) for multiple pair-wise comparisons (one-way ANOVA with Bonferroni’s *post hoc* tests)

Andrographolide (>98 % purity, Fig. 1a), LPS from *Salmonella enterica*, as well as other reagent grade chemicals were purchased from Sigma-Aldrich Ltd. (St. Louis, MO, USA) unless otherwise specified. Animals were obtained from the Centre for Animal Resources, National University of Singapore (NUS), and housed in ventilated cages in the NUS vivarium on a 12-h light/12-h dark cycle, with ad libitum access to water and standard chow. The study has been approved by the Institutional Animal Care and Use Committee at NUS (S13-5925, S13-6210 and NUS127/08) and followed the ARRIVE guidelines of the National Centre for the Replacement Refinement and Reduction of Animals in Research. ICR mice (male, 20 g ± 3 g) were randomly selected, then subjected to three intraperitoneal (i.p.) injection of LPS (3 mg/kg) over 24 h, together with oral gavage of vehicle (polyethylene glycol, PEG, 100 μL) or andrographolide (25 or 50 mg/kg) 1 h after each injection, based on slight modifications of a previously published regimen [32] (see Fig. 1b). Control mice were injected i.p. with phosphate-buffered saline and orally administered PEG on the same regimen. The mice were sacrificed 4 h after the last andrographolide gavage, and the brains were dissected free of the meninges and cerebellum, followed by homogenization in ice-cold buffer (50 mM Tris-HCl, 120 mM NaCl, 5 mM KCl, 2 μg/mL pepstatin A, supplemented with Complete ULTRA™ protease inhibitor tablets and PhosSTOP™ phosphatase inhibitor from Roche Life Science, Penzberg, Germany) using a Ultra-Turrax® handheld homogenizer (IKA-Werke, Staufen im Breisgau, Germany). Cortical homogenates were agitated on ice in an ultrasonicator for 40 min, then centrifuged at 6000×*g* for 20 min at 4 °C. The resultant supernatant was aliquoted and stored at −80 °C. Primary astrocyte cultures for in vitro assays were obtained from newborn Sprague Dawley rat pups (postnatal days 1–3) as previously described [24].

### Chemokine measurements in mouse cortical homogenates

Supernatants from the mouse cortical homogenates (see above) were thawed, vortexed, and measured for total protein (Pierce™ Coomassie assay, ThermoFisher Scientific, Waltham, MA, USA). The chemokines CCL-2, CCL-5, CXCL-1, CXCL-2, CXCL-9, and CXCL-10 were measured in by Luminex xMAP®-based assays according to the manufacturer’s instructions (Merck Millipore, Darmstadt, Germany). The cortical chemokine concentrations were calculated based on the standard curves generated, and expressed in pg/mg total brain protein.

### Cell viability assays

The rat primary astrocytes were plated onto 24-well tissue culture plates at a density of 1 × 10^5^ cells per well and treated with various concentrations of andrographolide (0–100 μM) or LPS (0–1000 ng/mL) for 48 h. Cell concentrations and viability were determined with the Muse™ Cell Analyzer (Merck Millipore, Darmstadt, Germany) according to the manufacturer’s instructions, and the percentages of viable cells were recorded.

### RT-PCR

For measurements of chemokine expression, the treated primary astrocytes were lysed in TRIzol® reagent (Thermo Fisher Scientific, Waltham, MA, USA), then processed for RNA isolation according to the manufacturer’s instructions (NucleoSpin® RNA kit, Macherey-Nagel, Düren, Germany). The concentration and purity of RNA were assessed using a NanoDrop spectrophotometer (Thermo Fisher Scientific, Waltham, MA, USA). Complementary DNA (cDNA) was synthesized from RNA samples using a high-capacity cDNA reverse transcriptase kit (Thermo Fisher Scientific, Waltham, MA, USA), and semi-quantitative real-time reverse transcription polymerase chain reaction (RT-PCR) was performed using GoTaq® qPCR Master Mix (Promega, Fitchburg, WI, USA) on an Applied Biosystems® StepOnePlus™ Real-Time PCR System (Thermo Fisher Scientific, Waltham, MA, USA). The primer sequences of the genes of interest are listed in Table 1, and results were normalized against the geometric mean of glyceraldehyde 3-phosphate dehydrogenase (GAPDH) and β-actin. Fold-change values of gene expression relative to control were computed for each experimental group using the 2^−ΔΔCT^ formula.

**Table 1:** RT-PCR primer sequences used in this study

Gene	Forward primer	Reverse primer
CCL2	5′-ATGCAGTTAATGCCCCACTC-3′	5′-TTCCTTATTGGGGTCAGCAC-3′
CCL5	5′-CCTTGCAGTCGTCTTTGTCA-3′	5′-ATCCCCAGCTGGTTAGGACT-3′
CXCL1	5′-GCGGAGAGATGAGAGTCTGG-3′	5′-AGGCATTGTGCCCTACAAAC-3′
CXCL5	5′-CGCTAATTTGGAGGTGATCC-3′	5′-AGTGCATTCCGCTTTGTTTT-3′
CXCL10	5′-GCTTATTGAAAGCGGTGAGC-3′	5′-GGTCAGGAGAAACAGGGACA-3′
CX3CL1	5′-CGCTCTGAATAGCTCCAACC-3′	5′-CTGCTCCTCAGGCCTACAAC-3′
TNF-α	5′-CCCAGACCCTCACACTCAGAT-3′	5′-TTGTCCCTTGAAGAGAACCTG-3′
β-actin	5′-ACCCGCGAGTACAACCTTCT-3′	5′-TTCTGACCCATACCCACCAT-3′
GAPDH	5′-CTCATGACCACAGTCCATGC-3′	5′-TTCTGACCCATACCCACCAT-3′

### Immunoblotting

Treated primary astrocytes were lysed in situ by adding a boiling Laemmli sample buffer (Bio-Rad, Berkeley, CA, USA) onto the tissue culture plates, and resultant lysates were further heated at 95 °C for 5 min. The samples were electrophoretically separated on 10 % polyacrylamide gels, transferred onto nitrocellulose membranes (Thermo Fisher Scientific, Waltham, MA, USA), and blocked with 5 % non-fat milk in 10 mM phosphate-buffered saline, pH 7.4 with 0.1 % Tween® 20 (PBST) at room temperature for 1 h. The membranes were then washed and probed with primary antibody diluted in PBST with 5 % bovine serum albumin overnight at 4 °C. The primary antibodies used were rabbit monoclonal phospho-p65 (pSer^536^, clone 93H1), p65 (clone D14E12), phospho-JNK (pThr^183^/pTyr^185^, clone 81E11), and rabbit polyclonal JNK, all at 1:1000 dilution (Cell Signaling Technology, Danvers, MA, USA). After primary antibody incubation, the membranes were washed with PBST, then incubated with respective horse radish peroxidase conjugated secondary antibodies (goat anti-rabbit, 1:5000; Jackson ImmunoResearch, West Grove, PA, USA) for 1 h at room temperature. The membranes were first probed for phospho-proteins then stripped and re-blotted for total proteins. Immunoblots were visualized using a horseradish peroxidase (HRP) substrate (Luminata™ Forte or Crescendo, Merck Millipore, Darmstadt, Germany) and quantified by an image analyzer (UVItec Ltd., Cambridge, UK).

### Statistical analyses

The results were reported as mean ± SEM, with *N* values representing individual animals or independent cell-based assays listed in the respective figure legends, and the data analyses were performed using SPSS Statistics software (version 21, IBM Inc., USA). Dose effects of andrographolide and LPS were compared to the untreated controls using analysis of variance (ANOVA) with Dunnett’s *post hoc* tests, while other pair-wise comparisons of the experimental groups were performed using ANOVA followed by Bonferroni’s post hoc tests, with *p* values <0.05 considered statistically significant.

## Results

### Effects of oral andrographolide on peripheral LPS-induced chemokines in the cortex

To assess potential therapeutic effects of andrographolide on neuroinflammatory conditions, a peripheral LPS administration model was selected as it rapidly induces brain inflammatory responses and may be pathophysiologically more relevant to bacterial meningitis or encephalitis-associated neuroinflammation, whose infectious source often invade the brain parenchyma from the periphery [33–35]. Similarly, we focused our studies on the cortex as it is considered most disease-relevant for neuroinflammatory conditions such as encephalitis. As shown in Fig. 1c, the mice subjected to three intraperitoneal injections of LPS over 24 h had significantly increased cortical levels of C-C (CCL-2, CCL-5) and C-X-C (CXCL-1, CXCL-2, CXCL-9, CXCL-10) chemokines, while LPS-treated animals administered orally with andrographolide 1 h after each LPS injection showed reductions of all measured chemokines except for CXCL10. Furthermore, the effects of andrographolide on LPS-induced chemokines also appeared to be dose-dependent, with 50 mg/kg showing more extensive reductions compared to 25 mg/kg in all responsive chemokines except CXCL9 (Fig. 1c). In the case of CCL2, CXCL1, and CXCL2, reductions only reached statistical significance with the higher andrographolide dose. Taken together, these results indicate that oral andrographolide administered soon after peripheral LPS is efficacious in attenuating a range of LPS-induced chemokines in the cortex.

### Effects of andrographolide on LPS-induced chemokine up-regulation in astrocytes

**Fig. 2:**
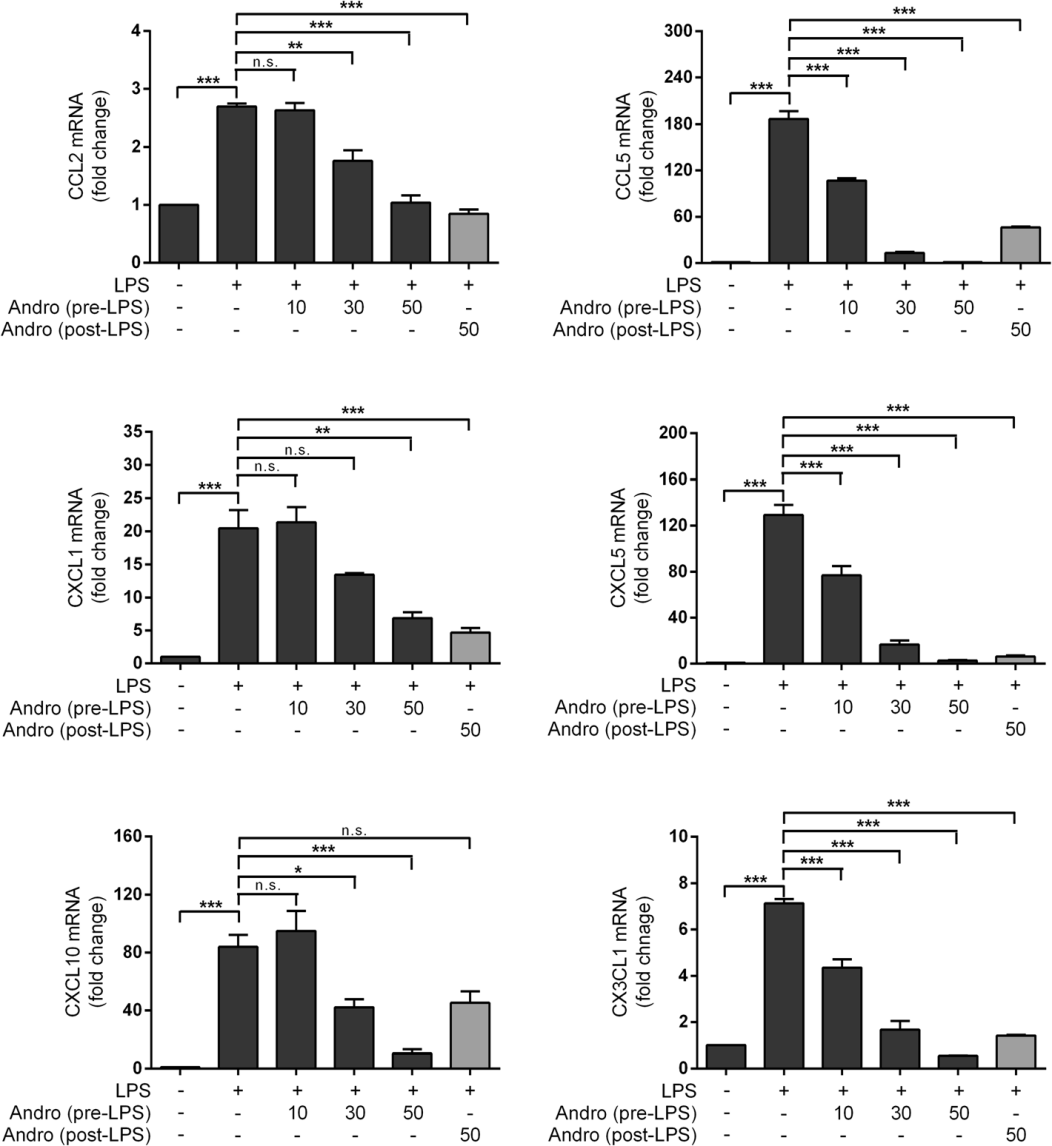
Andrographolide attenuated LPS-induced chemokine mRNA up-regulation in astrocytes. Various concentrations of andrographolide (in μM) were added to rat primary astrocytes in culture media 4 h before (“pre-LPS”) or 4 h after LPS (100 ng/mL) stimulation (“post-LPS"), then incubated for different periods such that the total incubation time with LPS was 12 h for both “pre-LPS” and “post-LPS” groups, before processing for RT-PCR. *Bar graphs* are mean ± SEM transcript fold-changes, with control levels set at 1 for *N* = 3 independent assays. All raw transcript values have been normalized to mean expression of housekeeping genes (see Methods) prior to conversion to fold-change values. *p < 0.05; **p < 0.01; ***p < 0.001. *n.s.* not significant (*p* > 0.05) for multiple pair-wise comparisons (one-way ANOVA with Bonferroni’s *post hoc* tests)

Although all brain cells respond to pro-inflammatory signals, microglia and astrocytes are known as the primary cell types mediating both the initiation and maintenance of neuroinflammation [6, 31]. In the in vitro studies, we focused on the potential involvement of astrocytes in mediating the chemokine responses to LPS as well as the effects of andrographolide on these responses and first investigated possible toxicity of LPS and andrographolide doses on cultured rat primary astrocytes. As shown in Additional file 1: Figure S1, astrocyte viability was not significantly altered by treatment with up to 100 μM andrographolide or 1000 ng/mL LPS for 48 h (*p* > 0.1, ANOVA with Dunnett’s *post hoc* tests). Similar to the mice cortical studies, LPS treatment induced transcription of messenger RNAs (mRNAs) for various chemokines from the C-C (CCL2, CCL5) and C-X-C (CXCL1, CXCL5, CXCL10) subfamilies in astrocytes (Fig. 2). To investigate potential effects of LPS on other chemokines, we included the C-X3-C member, CX3CL1 (not included in the Luminex-based kits for mice) and showed that it was also up-regulated by LPS (Fig. 2). The observed changes in all measured chemokines were attenuated dose-dependently by andrographolide pre-incubation, albeit with variable efficacy (Fig. 2). Importantly, we showed that andrographolide added 4 h after LPS treatment still resulted in attenuation of the LPS-induced up-regulation in all measured chemokines except CXCL10 (see Fig. 2, “Andro (post-LPS)” bars), suggesting the potential therapeutic utility of andrographolide after acute onset of disease.

**Fig. 3:**
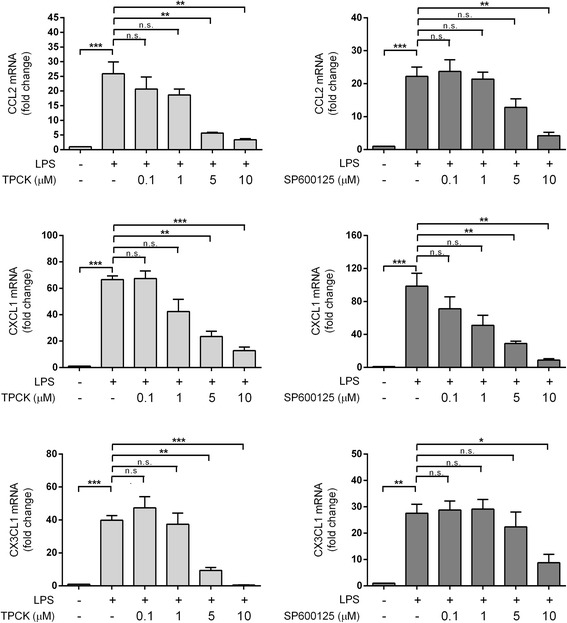
Inhibitors of NF-κB or JNK attenuated LPS-induced chemokine mRNA up-regulation in astrocytes. Various concentrations of TPCK (NF-κB inhibitor, *left panels*) or SP600125 (JNK inhibitor, *right panels*) were added to rat primary astrocytes in culture media 1 h before LPS stimulation (100 ng/mL), then incubated for a further 12 h before processing for RT-PCR. *Bar graphs* are mean ± SEM transcript fold-changes, with control levels set at 1 for *N* = 3 independent assays. All raw transcript values have been normalized to mean expression of housekeeping genes (see Methods) prior to conversion to fold-change values. *p < 0.05; **p < 0.01; ***p < 0.001. *n.s.* not significant (*p* > 0.05) for multiple pair-wise comparisons (one-way ANOVA with Bonferroni’s post hoc tests)

Given the well-established roles of NF-κB and JNK in regulating the expression of multiple inflammatory mediators, including the chemokines under study [27, 28, 36–38], we hypothesized that the observed LPS-induced chemokine gene up-regulation are dependent on NF-κB and JNK activation. Figure 3 shows that pre-incubation with a NF-κB (TPCK [39]) or JNK (SP600125 [40]) inhibitor was indeed able, with variable efficacy, to attenuate LPS-induced up-regulation of representative members of the C-C (CCL2), C-X-C (CXCL1) and C-X3-C (CX3CL1) chemokines.

### Effects of andrographolide on LPS-induced TNF-α up-regulation in astrocytes

**Fig. 4:**
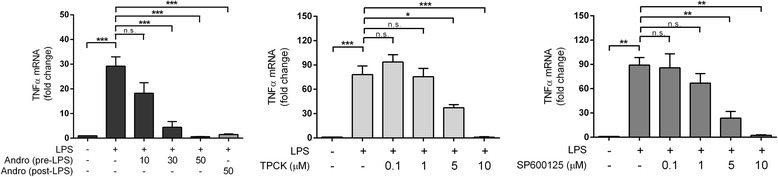
Andrographolide as well as inhibitors of NF-κB or JNK attenuated LPS-induced TNF-α mRNA up-regulation in astrocytes. Various concentrations of andrographolide, TPCK, and SP600125 were added to rat primary astrocytes with LPS stimulation as described in the figure legends of Figs. 2 and 3, before the treated cells were processed for RT-PCR. *Bar graphs* are mean ± SEM transcript fold-changes, with control levels set at 1 for *N* = 3 independent assays. All raw transcript values have been normalized to mean expression of housekeeping genes (see Methods) prior to conversion to fold-change values. *p < 0.05; **p < 0.01; ***p < 0.001. *n.s.* not significant (*p* > 0.05) for multiple pair-wise comparisons (one-way ANOVA with Bonferroni’s *post hoc* tests)

While the binding of LPS to toll-like receptor 4 rapidly activates NF-κB which in turn induces cytokine and chemokine expression [36], LPS is also known to stimulate the release of TNF-α [41–43], an acute phase protein that is rapidly elevated in response to injury or infection [44, 45]. In Fig. 4, we show that LPS-induced up-regulation of TNF-α mRNA could be attenuated by andrographolide, as well as by TPCK or SP600125, again suggesting that in astrocytes, andrographolide’s attenuative action on LPS-induced TNF-α up-regulation may be mediated in part by the inhibition of NF-κB and JNK.

### Effects of LPS and andrographolide on NF-κB and JNK activation in astrocytes

**Fig. 5:**
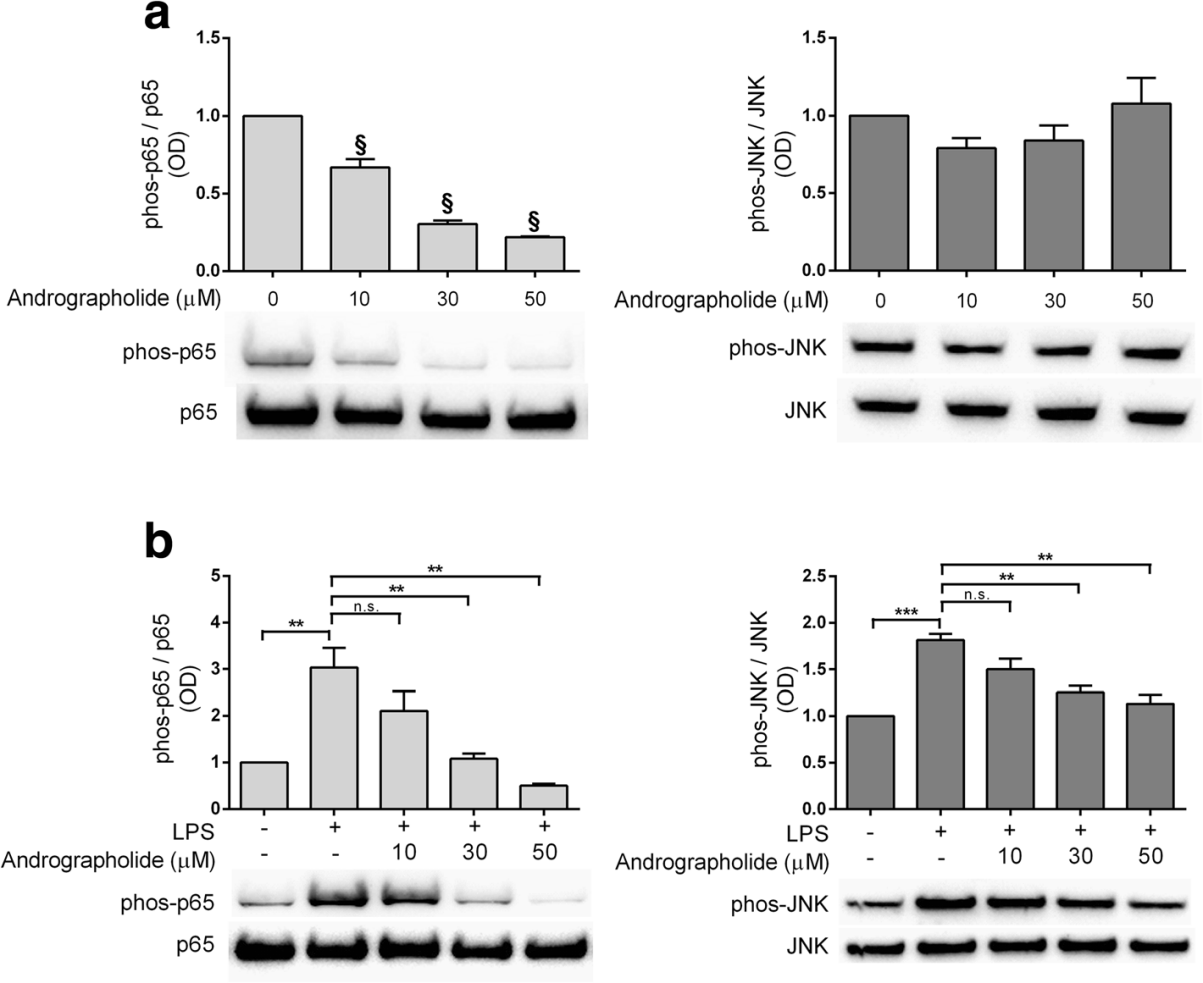
Andrographolide attenuated LPS-induced activation of NF-κB p65 and JNK in astrocytes. **a** Effects of andrographolide on basal activation of NF-κB and JNK (indicated by immunoreactivities of phospho-p65 and phospho-JNK, respectively, see *representative blots*). Primary astrocytes were treated for 6 h with various concentrations of andrographolide. **b** Effects of andrographolide on LPS activation of NF-κB and JNK (see *representative blots*). Primary astrocytes were pretreated with various concentrations of andrographolide for 4 h followed by 1-h LPS stimulation (100 ng/mL) in culture media. All *bar graphs* show immunoreactivities (mean ± SEM fold-changes in optical densities, OD, with untreated group set at 1) of phosphorylated proteins normalized to respective total proteins, *N* = 3–4 independent assays. ^§^ *p* < 0.001, significantly different from untreated controls (one-way ANOVA with Dunnett’s post hoc tests); **p < 0.01; ***p < 0.001. *n.s.* not significant (*p* > 0.05) for multiple pair-wise comparisons (one-way ANOVA with Bonferroni’s post hoc tests)

Finally, to validate andrographolide’s effects on NF-κB and JNK-mediated signaling which have been postulated to underlie the regulation of TNF-α and chemokines, we studied the effects of andrographolide on the activation of NF-κB and JNK, indicated, respectively, by p65 phosphorylated at Ser^536^ (phospho-p65) and JNK phosphorylated at Thr^183^/Tyr^185^ (phospho-JNK). Figure 5a showed that in the absence of LPS, andrographolide dose-dependently reduced phospho-p65 to below basal levels, while having no significant effect on basal phospho-JNK. In contrast, LPS (100 ng/mL) increased both phospho-p65 and phospho-JNK, effects which were dose-dependently attenuated by pre-incubation with andrographolide (Fig. 5b). Furthermore, neither LPS nor andrographolide appeared to affect total p65 and JNK immunoreactivities (Figs. 5a and b, bottom representative immunoblots), suggesting both LPS and andrographolide affect NF-κB- and JNK-mediated pathways via phosphorylation-dependent activation or inactivation rather than changes in NF-κB or JNK protein expression over the time-course under study.

These data, taken together with our earlier observations of attenuation of LPS-induced TNF-α and various chemokines (Figs. 3 and 4), suggest that andrographolide’s attenuation of LPS-induced chemokine up-regulation in astrocytes may be mediated, at least in part, by inhibition of NF-κB and JNK activation.

## Discussion

Chemokines are important mediators of neuroinflammatory processes, and dysregulation of chemokines is implicated in several diseases, thus making chemokines a viable target in anti-inflammation therapies [46]. To this end, we have confirmed the efficacy of oral andrographolide in regulating cortical chemokines from the C-C and C-X-C subfamilies in a peripherally administered LPS model. In separate experiments, we showed that andrographolide also attenuated LPS-induced gene up-regulation of a range of chemokines in cultured primary astrocytes. However, several differences exist in the two series of experiments, which may be due in part to the different species used (mice brain versus rat astrocytes), measurements of proteins versus mRNA, and variable levels and responses of chemokines. The selection of different species for the different experiments was due to logistical considerations, with mice being more easily manipulated and cost-effective for the animal studies, while rats, due to their larger brains, allowed higher cell yield in cell culture-based assays. Despite these limitations, our data, when considered *in toto*, suggest that oral andrographolide may have utility as an anti-neuroinflammatory, and that part of its mechanism of action may involve the regulation of astrocyte-derived chemokines. Additionally, our findings corroborate previous pharmacokinetic studies showing the ability of peripheral andrographolide to cross the blood-brain barrier (BBB) and reach the brain [47], especially during active neuroinflammation when BBB permeability is altered by cytokines and chemokines from the blood, brain, or brain endothelial cells [48, 49]. Further considerations of andrographolide’s therapeutic utility include its low toxicity, with LD_50_ by oral gavage of >4000 mg/kg/day [50]. Since our results indicated efficacy in regulating cortical chemokines at 150 mg/kg/day (three oral gavages of 50 mg/kg over 24 h), andrographolide will likely have a wide therapeutic window.

Given the efficacy of andrographolide in regulating a range of chemokines from the C-C, C-X-C, and C-X3-C (measured only in the in vitro assays) subfamilies, it is likely that andrographolide may influence a number of processes mediated by these chemokines. For example, CCL2 and CCL5 are potent chemoattractants for various leukocytes including granulocytes, monocytes, T lymphocytes, natural killer cells, and dendritic cells [51]. In experimental bacterial meningitis, CCL2 and CCL5 were found to be significantly elevated in cerebrospinal fluid (CSF), together with leukocyte infiltration [52], and antibodies neutralizing CCL2/CCL3 have been shown to mitigate neutrophil and macrophage recruitment into the brain [53], suggesting potential therapeutic efficacy for the chemokine-regulating andrographolide in bacterial meningitis. For C-X-C chemokines, besides the possible involvement of CXCL1, CXCL2, and CXCL5 in promoting leukocyte transmigration during bacterial meningitis [52, 54], CXCL1 and CXCL2 are known to be rapidly secreted by astrocytes in spinal cord injury, and attenuating these chemokines was associated with reduced neuronal death and improved motor function recovery [55, 56]. Detrimental effects of CXCL5 have also been reported in ischemic stroke, where CXCL5 elevation in CSF was associated with brain infarct size [57]. Similarly, although CXCL9 and CXCL10 expression are vital to the host’s response to CNS viral infections [58], persistent increases in astrocyte secretion of CXCL10 correlated with increased disease severity [59], while CXCL10 neutralization improved neurological outcome [60]. Finally, the only member of C-X3-C subfamily, CX3CL1, is recognized by CX3CR1 receptors preferentially expressed in microglia, indicating an essential role of CX3CL-1 in regulating microglial-mediated chronic neuroinflammation [61]. Attenuation of CX3CL1 signaling by CX3CL1 or CX3CR1 gene knockdown both significantly reduced infarct size, expression of IL-1β and TNF-α, and leukocyte infiltration in ischemic stroke models [62, 63]. In the current study, we showed that astrocytes up-regulate CX3CL1 expression upon LPS stimulation (Fig. 3), implying the potential for crosstalk between activated astrocytes and microglia. There is growing evidence that this crosstalk can lead to amplification of inflammatory responses [31, 64]. Inhibition of induced CX3CL1 expression in astrocytes therefore has the potential to interfere with astrocyte-microglia crosstalk and control dysregulated microglial activation.

Besides chemokines, cytokines such as TNF-α are released acutely in response to injury or infection [44, 45], and function to initiate and maintain the inflammatory process in part by stimulating other pro-inflammatory cytokines (e.g., IL-1α, IL-1β, and IL-6 [65, 66]) and chemokines (e.g., CCL2, CCL3, and CXCL2 [67, 68]). Therefore, our study suggests two pathways by which andrographolide attenuate LPS-induced chemokines, a direct pathway targeting LPS induction of chemokines and an indirect one via LPS-induced TNF-α. Interestingly, both pathways seem to be dependent on NF-κB and JNK activities (Figs. 3 and 4), and andrographolide’s mechanism of inhibitory action seems to be mediated in part by inactivation of these signaling molecules (Fig. 5). Furthermore, our data suggest that andrographolide may act synergistically with other potential anti-neuroinflammatory therapeutics such as selective estrogen receptor modulators [69, 70].

## Conclusions

**Fig. 6:**
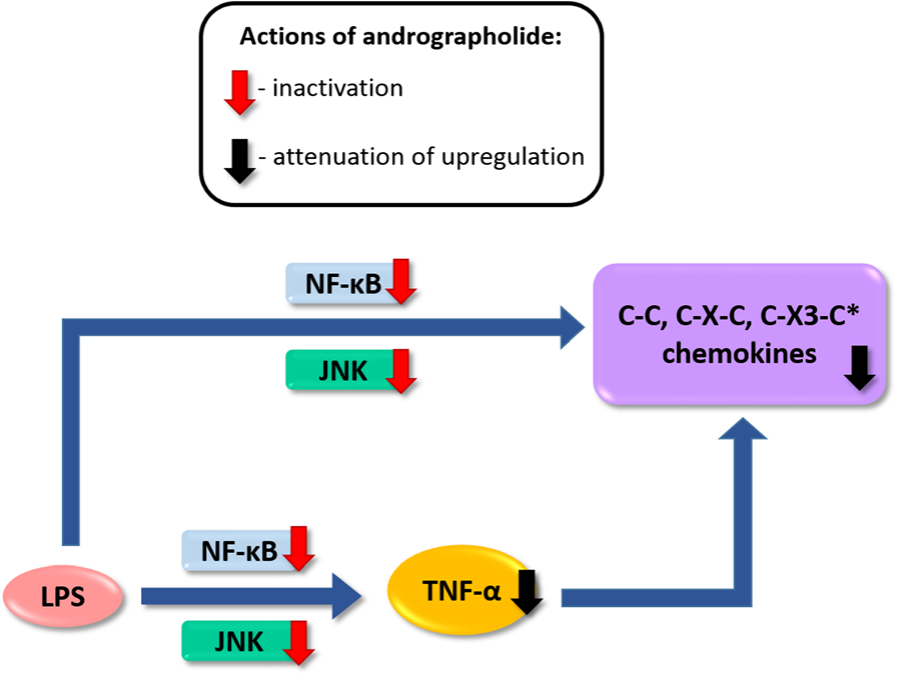
Putative pathways underlying andrographolide’s anti-neuroinflammatory effects following LPS stimulation. A schematic showing the effects of andrographolide on LPS-stimulated chemokines as well as TNF-α, and the putative signaling pathways underlying these effects. *C-X3-C subfamily has only one known member, CX3CL1 which was measured only in the in vitro experiments with rat primary astrocytes

In summary, we showed in a peripheral LPS-treated mouse model and in rat primary astrocytes that andrographolide has efficacy in abrogating LPS induction of chemokine up-regulation, in part by inactivation of NF-κB and JNK (Fig. 6). This suggests the potential utility of andrographolide as a therapeutic for neuroinflammatory conditions, especially those characterized by dysregulation of astrocyte-derived chemokines [8–11]. However, further work is needed to (i) characterize the effects of andrographolide in other brain cell types, e.g., neurons and microglia; (ii) better understand the dose-response relationships (for e.g., would there be more significant effects on CXCL10 (Figs. 1 and 2) with higher andrographolide doses?); (iii) confirm the behavioral correlates of chemokine regulation (for e.g., does inflammation-associated sickness behavior or cognitive impairments [34, 61] improve?); and (iv) elucidate other molecular pathways underlying andrographolide actions in the CNS (for e.g., involvement of mitogen-activated protein kinase-mediated signaling pathways [71] or with other components of signaling complexes like NF-κB p50 subunit [72]). It is expected that these studies will help further assess the feasibility of using andrographolide as an anti-neuroinflammatory therapeutic.

## Additional file

Additional file 1: Figure S1. Supplementary Fig. 1. Cell viability of primary astrocytes (in mean ± S.E.M. % of untreated) after 48 h incubation with andrographolide and LPS at the indicated concentrations. (ESM 210 kb)

## Abbreviations

ANOVA: analysis of variance
BBB: blood-brain barrier
CCL: C-C motif ligand
CNS: central nervous system
CX3CL: C-X3-C motif ligand
CXCL: C-X-C motif ligand
GAPDH: glyceraldehyde 3-phosphate dehydrogenase
HRP: horseradish peroxidase
IL-1β: interleukin-1β
JNK: c-Jun N-terminal kinase
LPS: lipopolysaccharide
NF-κB: nuclear factor-κB
PBST: phosphate-buffered saline with 0.1 % Tween® 20
PEG: polyethylene glycol
RT-PCR: reverse transcription polymerase chain reaction
TNF-α: tumor necrosis factor-α

## References

[1] Mennicken F, Maki R, de Souza EB, Quirion R (1999). Chemokines and chemokine receptors in the CNS: a possible role in neuroinflammation and patterning. Trends Pharmacol Sci.

[2] Adler MW, Rogers TJ (2005). Are chemokines the third major system in the brain?. J Leukoc Biol.

[3] Padovani-Claudio DA, Liu L, Ransohoff RM, Miller RH (2006). Alterations in the oligodendrocyte lineage, myelin, and white matter in adult mice lacking the chemokine receptor CXCR2. Glia.

[4] Tsai HH, Frost E, To V, Robinson S, Ffrench-Constant C, Geertman R (2002). The chemokine receptor CXCR2 controls positioning of oligodendrocyte precursors in developing spinal cord by arresting their migration. Cell.

[5] de Haas AH, van Weering HR, de Jong EK, Boddeke HW, Biber KP (2007). Neuronal chemokines: versatile messengers in central nervous system cell interaction. Mol Neurobiol.

[6] Ransohoff RM, Brown MA (2012). Innate immunity in the central nervous system. J Clin Invest.

[7] Sofroniew MV (2009). Molecular dissection of reactive astrogliosis and glial scar formation. Trends Neurosci.

[8] Gyoneva S, Ransohoff RM (2015). Inflammatory reaction after traumatic brain injury: therapeutic potential of targeting cell-cell communication by chemokines. Trends Pharmacol Sci.

[9] Kerstetter AE, Padovani-Claudio DA, Bai L, Miller RH (2009). Inhibition of CXCR2 signaling promotes recovery in models of multiple sclerosis. Exp Neurol.

[10] Liu C, Cui G, Zhu M, Kang X, Guo H (2014). Neuroinflammation in Alzheimer’s disease: chemokines produced by astrocytes and chemokine receptors. Int J Clin Exp Pathol.

[11] Mastroianni CM, Lancella L, Mengoni F, Lichtner M, Santopadre P, D’Agostino C (1998). Chemokine profiles in the cerebrospinal fluid (CSF) during the course of pyogenic and tuberculous meningitis. Clin Exp Immunol.

[12] Chao WW, Lin BF (2010). Isolation and identification of bioactive compounds in Andrographis paniculata (Chuanxinlian). Chin Med.

[13] Panossian A, Davtyan T, Gukassyan N, Gukasova G, Mamikonyan G, Gabrielian E (2002). Effect of andrographolide and Kan Jang—fixed combination of extract SHA-10 and extract SHE-3—on proliferation of human lymphocytes, production of cytokines and immune activation markers in the whole blood cells culture. Phytomedicine.

[14] Arifullah M, Namsa ND, Mandal M, Chiruvella KK, Vikrama P, Gopal GR (2013). Evaluation of anti-bacterial and anti-oxidant potential of andrographolide and echiodinin isolated from callus culture of Andrographis paniculata Nees. Asian Pac J Trop Biomed.

[15] Chua LS (2014). Review on liver inflammation and antiinflammatory activity of Andrographis paniculata for hepatoprotection. Phytother Res.

[16] Guan S, Tee W, Ng D, Chan T, Peh H, Ho W (2013). Andrographolide protects against cigarette smoke-induced oxidative lung injury via augmentation of Nrf2 activity. Br J Pharmacol.

[17] Lim JC, Chan TK, Ng DS, Sagineedu SR, Stanslas J, Wong WS (2012). Andrographolide and its analogues: versatile bioactive molecules for combating inflammation and cancer. Clin Exp Pharmacol Physiol.

[18] Sheeja K, Kuttan G (2006). Protective effect of Andrographis paniculata and andrographolide on cyclophosphamide-induced urothelial toxicity. Integr Cancer Ther.

[19] Lipinski CA, Lombardo F, Dominy BW, Feeney PJ (2001). Experimental and computational approaches to estimate solubility and permeability in drug discovery and development settings. Adv Drug Deliv Rev.

[20] Amaryan G, Astvatsatryan V, Gabrielyan E, Panossian A, Panosyan V, Wikman G (2003). Double-blind, placebo-controlled, randomized, pilot clinical trial of ImmunoGuard--a standardized fixed combination of Andrographis paniculata Nees, with Eleutherococcus senticosus Maxim. Schizandra chinensis Bail. and Glycyrrhiza glabra L. extracts in patients with Familial Mediterranean Fever. Phytomedicine.

[21] Burgos RA, Hancke JL, Bertoglio JC, Aguirre V, Arriagada S, Calvo M (2009). Efficacy of an Andrographis paniculata composition for the relief of rheumatoid arthritis symptoms: a prospective randomized placebo-controlled trial. Clin Rheumatol.

[22] Chan SJ, Wong WS, Wong PT, Bian JS (2010). Neuroprotective effects of andrographolide in a rat model of permanent cerebral ischaemia. Br J Pharmacol.

[23] Tzeng YM, Lee YC, Cheng WT, Shih HN, Wang HC, Rao YK (2012). Effects of andrographolide and 14-deoxy-11,12-didehydroandrographolide on cultured primary astrocytes and PC12 cells. Life Sci.

[24] Wong SY, Chan SJ, Wong WS, Wong PT, Lai MK (2014). Andrographolide attenuates interleukin-1b-stimulated upregulation of chemokine CCL5 and glial fibrillary acidic protein in astrocytes. Neuroreport.

[25] Chen YY, Hsu MJ, Hsieh CY, Lee LW, Chen ZC, Sheu JR (2014). Andrographolide inhibits nuclear factor-κB activation through JNK-Akt-p65 signaling cascade in tumor necrosis factor-α-stimulated vascular smooth muscle cells. ScientificWorldJournal.

[26] Ji L, Shen K, Jiang P, Morahan G, Wang Z (2011). Critical roles of cellular glutathione homeostasis and jnk activation in andrographolide-mediated apoptotic cell death in human hepatoma cells. Mol Carcinog.

[27] Wang LW, Tu YF, Huang CC, Ho CJ (2012). JNK signaling is the shared pathway linking neuroinflammation, blood–brain barrier disruption, and oligodendroglial apoptosis in the white matter injury of the immature brain. J Neuroinflammation.

[28] Wang LW, Chang YC, Chen SJ, Tseng CH, Tu YF, Liao NS (2014). TNFR1-JNK signaling is the shared pathway of neuroinflammation and neurovascular damage after LPS-sensitized hypoxic-ischemic injury in the immature brain. J Neuroinflammation.

[29] Paul D, Ge S, Lemire Y, Jellison ER, Serwanski DR, Ruddle NH (2014). Cell-selective knockout and 3D confocal image analysis reveals separate roles for astrocyte-and endothelial-derived CCL2 in neuroinflammation. J Neuroinflammation.

[30] Pekny M, Pekna M (2014). Astrocyte reactivity and reactive astrogliosis: costs and benefits. Physiol Rev.

[31] Sofroniew MV, Vinters HV (2010). Astrocytes: biology and pathology. Acta Neuropathol.

[32] Erickson MA, Banks WA (2011). Cytokine and chemokine responses in serum and brain after single and repeated injections of lipopolysaccharide: multiplex quantification with path analysis. Brain Behav Immun.

[33] Kastenbauer S, Winkler F, Fesl G, Schiel X, Ostermann H, Yousry TA (2001). Acute severe spinal cord dysfunction in bacterial meningitis in adults: MRI findings suggest extensive myelitis. Arch Neurol.

[34] Konsman JP, Parnet P, Dantzer R (2002). Cytokine-induced sickness behaviour: mechanisms and implications. Trends Neurosci.

[35] Skelly DT, Hennessy E, Dansereau MA, Cunningham C (2013). A systematic analysis of the peripheral and CNS effects of systemic LPS, IL-1β, TNF-α and IL-6 challenges in C57BL/6 mice. PLoS One.

[36] Hayden MS, West AP, Ghosh S (2006). NF-κB and the immune response. Oncogene.

[37] Li M, Wu ZM, Yang H, Huang SJ (2011). NFκB and JNK/MAPK activation mediates the production of major macrophage- or dendritic cell-recruiting chemokine in human first trimester decidual cells in response to proinflammatory stimuli. J Clin Endocrinol Metab.

[38] Wuyts WA, Vanaudenaerde BM, Dupont LJ, Demedts MG, Verleden GM (2003). Involvement of p38 MAPK, JNK, p42/p44 ERK and NF-κB in IL-1β-induced chemokine release in human airway smooth muscle cells. Respir Med.

[39] Ha KH, Byun MS, Choi J, Jeong J, Lee KJ, Jue DM (2009). N-tosyl-L-phenylalanine chloromethyl ketone inhibits NF-kB activation by blocking specific cysteine residues of IkB kinase b and p65/RelA. Biochemistry.

[40] Bennett BL, Sasaki DT, Murray BW, O’Leary EC, Sakata ST, Xu W (2001). SP600125, an anthrapyrazolone inhibitor of Jun N-terminal kinase. Proc Natl Acad Sci U S A.

[41] Agbanoma G, Li C, Ennis D, Palfreeman AC, Williams LM, Brennan FM (2012). Production of TNF-α in macrophages activated by T cells, compared with lipopolysaccharide, uses distinct IL-10-dependent regulatory mechanism. J Immunol.

[42] Guha M, Mackman N (2001). LPS induction of gene expression in human monocytes. Cell Signal.

[43] Zipper LM, Mulcahy RT (2000). Inhibition of ERK and p38 MAP kinases inhibits binding of Nrf2 and induction of GCS genes. Biochem Biophys Res Commun.

[44] Kaminska B, Gozdz A, Zawadzka M, Ellert‐Miklaszewska A, Lipko M (2009). MAPK signal transduction underlying brain inflammation and gliosis as therapeutic target. Anat Rec.

[45] van Miert AS (1995). Pro-inflammatory cytokines in a ruminant model: pathophysiological, pharmacological, and therapeutic aspects. Vet Q.

[46] Pease JE, Williams TJ (2006). The attraction of chemokines as a target for specific anti-inflammatory therapy. Br J Pharmacol.

[47] Bera R, Ahmed SK, Sarkar L, Sen T, Karmakar S (2014). Pharmacokinetic analysis and tissue distribution of andrographolide in rat by a validated LC-MS/MS method. Pharm Biol.

[48] Banks WA, Erickson MA (2010). The blood–brain barrier and immune function and dysfunction. Neurobiol Dis.

[49] Verma S, Nakaoke R, Dohgu S, Banks WA (2006). Release of cytokines by brain endothelial cells: A polarized response to lipopolysaccharide. Brain Behav Immun.

[50] Chen JX, Xue HJ, Ye WC, Fang BH, Liu YH, Yuan SH (2009). Activity of andrographolide and its derivatives against influenza virus in vivo and in vitro. Biol Pharm Bull.

[51] Jaerve A, Müller HW (2012). Chemokines in CNS injury and repair. Cell Tissue Res.

[52] Hanisch UK, Prinz M, Angstwurm K, Häusler KG, Kann O, Kettenmann H (2001). The protein tyrosine kinase inhibitor AG126 prevents the massive microglial cytokine induction by pneumococcal cell walls. Eur J Immunol.

[53] Diab A, Abdalla H, Li HL, Shi FD, Zhu J, Höjberg B (1999). Neutralization of macrophage inflammatory protein 2 (MIP-2) and MIP-1α attenuates neutrophil recruitment in the central nervous system during experimental bacterial meningitis. Infect Immun.

[54] Zwijnenburg PJ, de Bie HM, Roord JJ, van der Poll T, van Furth AM (2003). Chemotactic activity of CXCL5 in cerebrospinal fluid of children with bacterial meningitis. J Neuroimmunol.

[55] Kang J, Jiang MH, Min HJ, Jo EK, Lee S, Karin M (2011). IKK-β-mediated myeloid cell activation exacerbates inflammation and inhibits recovery after spinal cord injury. Eur J Immunol.

[56] Pineau I, Sun L, Bastien D, Lacroix S (2010). Astrocytes initiate inflammation in the injured mouse spinal cord by promoting the entry of neutrophils and inflammatory monocytes in an IL-1 receptor/MyD88-dependent fashion. Brain Behav Immun.

[57] Zaremba J, Skrobański P, Losy J (2006). The level of chemokine CXCL5 in the cerebrospinal fluid is increased during the first 24 h of ischaemic stroke and correlates with the size of early brain damage. Folia Morphol.

[58] Thapa M, Welner RS, Pelayo R, Carr DJ (2008). CXCL9 and CXCL10 expression are critical for control of genital herpes simplex virus type 2 infection through mobilization of HSV-specific CTL and NK cells to the nervous system. J Immunol.

[59] Bhowmick S, Duseja R, Das S, Appaiahgiri MB, Vrati S, Basu A (2007). Induction of IP-10 (CXCL10) in astrocytes following Japanese encephalitis. Neurosci Lett.

[60] Liu MT, Keirstead HS, Lane TE (2001). Neutralization of the Chemokine CXCL10 reduces inflammatory cell invasion and demyelination and improves neurological function in a viral model of multiple sclerosis. J Immunol.

[61] Briones TL, Woods J, Wadowska M (2014). Chronic neuroinflammation and cognitive impairment following transient global cerebral ischemia: role of fractalkine/CX3CR1 signaling. J Neuroinflammation.

[62] Dénes Á, Ferenczi S, Halász J, Környei Z, Kovács KJ (2008). Role of CX3CR1 (fractalkine receptor) in brain damage and inflammation induced by focal cerebral ischemia in mouse. J Cereb Blood Flow Metab.

[63] Soriano SG, Amaravadi LS, Wang YF, Zhou H, Yu GX, Tonra JR (2002). Mice deficient in fractalkine are less susceptible to cerebral ischemia-reperfusion injury. J Neuroimmunol.

[64] Saijo K, Winner B, Carson CT, Collier JG, Boyer L, Rosenfeld MG (2009). A Nurr1/CoREST pathway in microglia and astrocytes protects dopaminergic neurons from inflammation-induced death. Cell.

[65] Kozawa O, Suzuki A, Kaida T, Tokuda H, Uematsu T (1997). Tumor necrosis factor-α autoregulates interleukin-6 synthesis via activation of protein kinase C: function of sphingosine 1-phosphate and phosphatidylcholine-specific phospholipase C. J Biol Chem.

[66] Turner NA, Mughal RS, Warburton P, O’Regan DJ, Ball SG, Porter KE (2007). Mechanism of TNFα-induced IL-1α, IL-1β and IL-6 expression in human cardiac fibroblasts: effects of statins and thiazolidinediones. Cardiovasc Res.

[67] Barna BP, Pettay J, Barnett GH, Zhou P, Iwasaki K, Estes ML (1994). Regulation of monocyte chemoattractant protein-1 expression in adult human non-neoplastic astrocytes is sensitive to tumor necrosis factor (TNF) or antibody to the 55-kDa TNF receptor. J Neuroimmunol.

[68] Czermak BJ, Sarma V, Bless NM, Schmal H, Friedl HP, Ward PA (1999). In vitro and in vivo dependency of chemokine generation on C5a and TNF-α. J Immunol.

[69] Arevalo MA, Diz-Chaves Y, Santos-Galindo M, Bellini MJ, Garcia-Segura LM (2012). Selective oestrogen receptor modulators decrease the inflammatory response of glial cells. J Neuroendocrinol.

[70] Cerciat M, Unkila M, Garcia-Segura LM, Arevalo MA (2010). Selective estrogen receptor modulators decrease the production of interleukin-6 and interferon-gamma-inducible protein-10 by astrocytes exposed to inflammatory challenge in vitro. Glia.

[71] Lu WJ, Lin KH, Hsu MJ, Chou DS, Hsiao G, Sheu JR (2012). Suppression of NF-κB signaling by andrographolide with a novel mechanism in human platelets: regulatory roles of the p38 MAPK-hydroxyl radical-ERK2 cascade. Biochem Pharmacol.

[72] Nguyen VS, Loh XY, Wijaya H, Wang J, Lin Q, Lam Y (2015). Specificity and inhibitory mechanism of andrographolide and its analogues as antiasthma agents on NF-kB p50. J Nat Prod.

