# Supplementary material for: Andrographolide attenuates LPS-stimulated up-regulation of C-C and C-X-C motif chemokines in rodent cortex and primary astrocytes
Source: J Neuroinflammation. 2016 Feb 9;13:34. doi: 10.1186/s12974-016-0498-6 (PMC4748554; doi:10.1186/s12974-016-0498-6)
Supplement: Additional file 1: Figure S1. — Supplementary Fig. 1. Cell viability of primary astrocytes (in mean ± S.E.M. % of untreated) after 48 h incubation with andrographolide and LPS at the indicated concentrations. (ESM 210 kb) [file 12974_2016_498_MOESM1_ESM.pdf]

Andrographolide attenuates LPS-stimulated up-regulation of C-C and C-X-C motif chemokines in rodent cortex and primary astrocytes

*Siew Ying Wong, Michelle G.K. Tan, William A. Banks, W.S. Fred Wong,
Peter T.-H. Wong, Mitchell K.P. Lai*

Supplementary Fig. 1

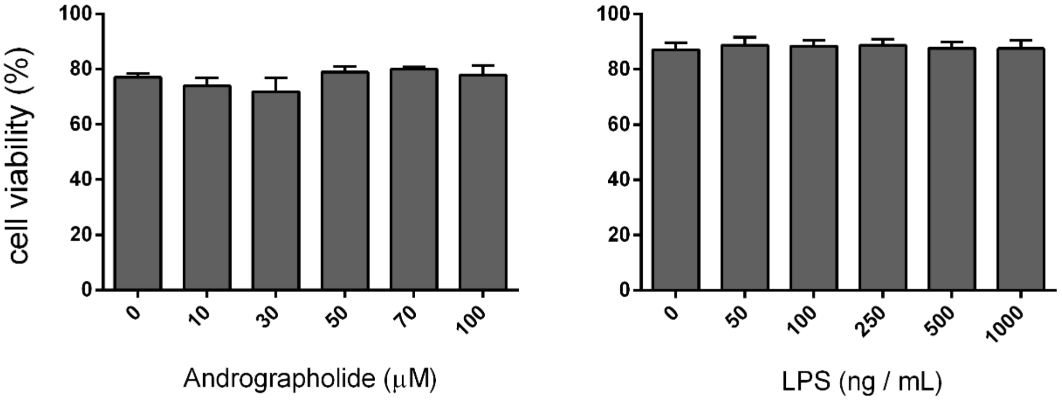

Cell viability of primary astrocytes (in mean ± S.E.M. % of untreated) after 48 h incubation with andrographolide and LPS at the indicated concentrations.
